# Indents

**Published:** 1909-10-15

**Authors:** 


### INDENTS.

**☞Brief Articles of Technical or Scientific Value will receive notice and comment. Contributions invited.**

*Cresol.* Cresol, a powerful germicide and a homologue of phenol, is about three times as powerful as the latter and when used in putrescent pulp canals is believed to unite chemically with the fats of decomposing proteid to form lysol, a good antiseptic.—Dr .E. B. Lodge in *Summary.*

*Calcium Fluorid in Dental Therapeutics.* If in a human tooth, which is composed of organic and calcareous elements, the organic elements predominate, that tooth is particularly predisposed to dental caries, while a predominance of the calcareous elements imparts to the tooth a certain degree of immunity. Scarcity of mineral salts in some teeth is due to physiological and pathological reasons.

In women, gestation and lactation deprive the osseous, nervous, and dental systems of a great many mineral elements, women therefore being less immune to caries, and sometimes falling victim to veritable catastrophes of the dental system. Besides these physiologic causes certain particular conditions of life favor dental decalcification. During the age of puberty in both sexes the daily diet is often not suited for supplying the system with the necessary amount of inorganic materials. Moreover, the strain imposed upon young people about the twenties, when they have to undergo the most important examinations that frequently determine their subsequent career, singularly coincides with the frequency of dental caries observed at that age.

The pathological causes embrace all diseases that more or less impede assimilation or produce dissimilation, *i.e.*

anemia of various intensity, tuberculosis in all its forms, and syphilis...

On this demineralization, Dr. Brissemoret writes in the *Bulletin General de Therapeutique*, July 30: "The fresh human bone contains from twenty-nine to fifty-seven per cent. of its own weight of mineral matter, and, after calcination, represents a mixture of chiefly tricalcic phosphate, together with phosphate of magnesia, calcium fluorid. The last plays a definite physiological part. It is a precious cement, which by uniting the particles of carbonate and calcium phosphate and magnesia, of which the animal skeleton is chiefly built up, imparts to it a durability and solidity which it would otherwise not possess. In order to assure the resistance of the enamel, nature accumulates mineral fluorids in these masticatory organs. Our organism eliminates every day an appreciable quantity of calcium fluorid, from 0.003 to 0.005 gram. Man takes in the necessary supply of these salts in his drinking water. If his diet does not restore the losses in fluorid, nutritional disturbances quickly arise. It is the lack of fluorid in the diet that constitutes one of the main causes of dental caries in its various forms." For such cases Dr. Brissemoret prescribes calcium fluorid. He reports one case of a young man whom he had under observation for several years, and who suffered from periodical attacks of caries. After the administration of 5 mgm. of calcium fluorid per day for two weeks a month, considerable improvement was noticed after six months' treatment. For children during the period of growth, for adults with dental caries, in tuberculosis, for stimulating the remineralization of the organism, in fractures for hastening the solidification of the callus, for women during gestation and lactation, he prescribes calcium florid in powder form as follows:

| | |
|---|---|
| Calcium fluorid - - - - - - | 0.075 gram |
| Potassium phosphate - - - - | 3 grams |
| Sodium phosphate - - - - - | 5 " |
| Magnesium phosphate - - - - | 10 " |
| Dicalcium phosphate - - - - - | 10 " |
| Sodium citrate - - - - - | 15 " |
| Lactose - - - - - - | q.s. ad 100 " |

One.half teaspoonful twice a day at meal-times.

For stimulating the formation of callus in fractures, the following formula is recommended:

| | | |
|---|---|---|
| Calcium fluorid | - - - - - | 0.05 gram |
| Magnesium fluorid | - - - - | 0.02 " |
| Calcium bromid | - - - - - | 2.50 grams |
| Dicalcium phosphate | - - - - | 5 " |
| Calcium carbonate | - - - - - - | 5 " |

In 20 powders. Two per day.

—*Cosmos.*

*The Work of Prof Dr. Jessen, of Strasburg.* Wherever the question of dental inspection in the schools is raised the name of Prof. Dr. Jessen, of Strasburg comes with it. He is the great originator of the work, and Strasburg is to-day the center of the movement for Europe. It is the center of an important work, for no less than thirty cities of the Fatherland support free dental clinics where work is done on the teeth of school children. Dr. Jessen became years ago impressed with the general diffidence prevalent among the people in the matter of dental hygiene, and came to the conclusion that a large percentage of illness and disease is due to defective teeth. He labored long and ardently in behalf of a better understanding on the part of the people, working as is natural through the medical fraternity. He has lived to see his views accepted by one continent with another just ready to fall in line; he has been able to found on them great gatherings of interested medical men. At the congress in London, two years ago, devoted to school hygiene, he met two thousand delegates from every part of the world, who had an opportunity to make themselves interested in his subject—the teeth of school children. In his own land he has seen a rare stirring on every hand. One result has been a close investigation by a State commision, which has reported on the condition of teeth in the schools and in the army. In brief, the conclusions of this body may thus be stated. That diseased teeth are the most common of all diseases; that the physical and mental development of children is injured by such teeth, the health suffering accordingly; that the improvement and eventually overcoming of these conditions is possible only by the introduction of dentists into the school and the army, and that it will be necessary to establish State dental hospitals, the cost of which will be small in comparison with the benefits received. For seven or eight years the work in Strasburg has been in progress under the eye of Dr. Jessen, so that what he has to say in the matter is born of actual experience. He himself conducted the smaller of the figurings with school children, those of the 20,000 scholars, and one may readily see that he has done his work upon a truly practical scale. His estimate for Strasburg have been that the cost is about twenty-five cents per pupil. The item of the health of the pupils has been very carefully looked into, and he asserts that there is evident improvement. The scholars show not only a benefit to health, but increased efficiency in their studies, and in addition save the time that was previously lost by them on account of ill health. So impressed is the municipality of Strasburg with the value of Jessen's work that it has erected, at a cost of $60,000, a building to be devoted to the service of the dental inspection of the children in the public schools. When one remembers the economy that Europeans have instilled into them from their earliest childhood, the expenditure of so considerable a sum is the best kind of proof of the high esteem in which the matter of dental hygiene in the schools is held.

Another outcome of the work of Prof. Jessen is that in Germany on the first day of February last past there was established a German Central Committee for the care of teeth in schools. The purpose of the association is to interest the people in the matter of the care of the teeth, to secure the publication and distribution of articles on the subject, to disseminate information among school associations, to have dental hygiene taught in the schools, and to enlist State and communal cooperation in forwarding the important work. This organization has already been the means of bringing into close touch for effective work on the same general plan the health officials and dentists for such cities as Berlin, Brandenburg, Breslau, Cologne, Heidelberg and Strasburg, together with prominent medical officers in the German army.—*Boston Transcript.*

